# Objective quantification of motion-induced dizziness using a proof-of-concept multimodal wearable platform

**DOI:** 10.1038/s41598-026-52443-5

**Published:** 2026-05-11

**Authors:** Nhan Cao, Brian Loyd, Andy Kittelson, Anh Nguyen

**Affiliations:** 1 Department of Computer Science, University of Montana, Missoula, MT 59812 USA; 2 School of Physical Therapy and Rehabilitation Science, University of Montana, Missoula, MT 59812 USA

**Keywords:** Engineering, Health care, Medical research, Neurology, Neuroscience

## Abstract

Vestibular dysfunction is a common cause of dizziness and a leading cause of medical visits. Yet, current assessment methods of dizziness remain largely subjective, relying on self-reports and intermittent clinical evaluations that lack real-time monitoring, quantitative precision, and preventive capability. This paper introduces EquilibriSense, a bio-inspired, head-worn system for quantifying motion-induced dizziness under a controlled head-rotation paradigm. The system integrates multiple physiological sensing modalities with an AI-driven pipeline and a neurocomputational labeling framework to model dizziness progression and classify symptom severity on a five-level scale (0-4). In a pilot study involving a small cohort of 10 healthy participants in a controlled laboratory setting, the system achieved 86.8% accuracy in multi-level motion-induced dizziness classification and enabled early detection of dizziness onset with an AUC of 0.99 and over 98% accuracy, supported by high precision and recall. These results demonstrate the feasibility of using multimodal physiological sensing to characterize motion-induced dizziness and establish EquilibriSense as a proof-of-concept platform for objective dizziness quantification, providing a foundation for future validation in real-world and clinical populations.

## Introduction

The human vestibular system, intricately located within the inner ear, plays a central role in maintaining balance, spatial orientation, and coordinated movement. According to the Vestibular Disorders Association, approximately 69 million Americans have experienced some form of vestibular dysfunction in their lifetime^1^. Common dysfunctions affecting this system include sensory conflict, Benign Paroxysmal Positional Vertigo (BPPV)^2^, Meniere’s disease^3^, vestibular neuronitis^4^, labyrinthitis^5^, and vestibular schwannoma^6^. These conditions often lead to a range of debilitating symptoms, with dizziness being the most prominent. Such symptoms can significantly disrupt daily activities, reduce mobility, and negatively impact overall life quality.

Several techniques exist to assess vestibular function, each targeting specific components of the system to aid in accurate diagnosis. Vestibular Evoked Myogenic Potential (VEMP), for example, are evoked responses resulting from vestibular stimulation of the otolithic organs using sound or vibration, providing information about otolith-mediated reflex pathways^7^. Other common tests include Electronystagmography (ENG)^8^, Videonystagmography (VNG)^9^, Video Head Impulse Test (vHIT)^10^, and Electrooculography (EOG)^11^, which monitor eye movements and assess the vestibulo-ocular reflex (VOR) using electrodes or video capture systems. Vestibular-Evoked Cerebral Potentials (VestEPs) employ electroencephalography (EEG) to examine cortical responses to vestibular stimuli, offering insights into both healthy function and neurological abnormalities^12,13^. Electrocochleography (ECoG), on the other hand, records electrical potentials near the eardrum in response to auditory stimuli and is particularly useful in identifying endolymphatic hydrops, a key indicator of Méniére’s disease^14,15^. Moreover, subjective assessments through standardized questionnaires provide complementary insights into symptom severity and functional impact^16–18^. While these traditional diagnostic methods are valuable, they are typically constrained to clinical environments, rely on periodic assessments, and often lack real-time or fully objective data, limiting their ability to capture the full extent of vestibular dysfunction in everyday settings.

**Fig. 1:**
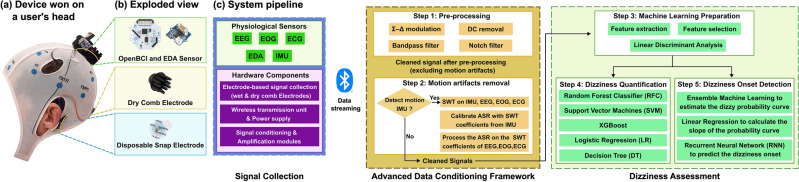
Overview of the EquilibriSense system. (**a**) Device placement and fit on a user’s head. (**b**) Exploded view of the hardware components. (**c**) End-to-end system pipeline from signal acquisition to dizziness assessment.

The rise of wearable technology has introduced promising avenues for advancing vestibular assessment beyond clinical settings. For instance, the Continuous Ambulatory Vestibular Assessment (CAVA) system^19,20^ is a reusable, ear-worn device with single-use facial electrodes that continuously tracks eye and head movements for up to thirty days to support clinicians in diagnosing dizziness and balance disorders. Another approach^21^ employs five synchronized inertial measurement units (IMUs) to analyze gait quality and stability in individuals with chronic vestibular hypofunction during dynamic tasks. The Walkasins™ device^22^ incorporates foot pressure sensors with leg-mounted vibratory feedback to deliver real-time tactile cues for individuals with peripheral neuropathy. Despite these advances, eye- and head-tracking systems primarily quantify external kinematics and visual-motor coordination, which may not fully reflect internal physiological responses to motion-induced dizziness. Similarly, IMU-based systems are effective for capturing gross movement patterns such as gait or postural sway, but provide limited information about underlying neural or autonomic state changes when external motion is constrained, such as during seated or stationary conditions. These limitations highlight the need for more comprehensive, real-time, and multi-symptom wearable systems capable of capturing physiological markers associated with dizziness-related responses across a range of contexts.

To address this gap, we present EquilibriSense, a lightweight, head-mounted wearable platform for quantification of motion-induced dizziness under a controlled active head-rotation paradigm. The system provides comprehensive physiological analysis using multimodal sensors, including EEG, EOG, electrocardiogram (ECG), electrodermal activity (EDA), and IMU. These sensors capture complementary physiological signals associated with motion-provoked discomfort. Integrated with signal processing and machine learning (ML) algorithms, EquilibriSense enables real-time detection of dizziness onset and classification of dizziness levels. Furthermore, we intentionally designed the system with minimal EEG and EOG configurations to support a discreet, deployable wearable form factor for convenient usage, demonstrating that meaningful physiological patterns can be extracted even under constrained sensing configurations. However, due to the physiologic complexity of the perception of motion-induced dizziness, realizing EquilibriSense encounters several challenges:

- Identifying informative physiological features associated with dizziness levels is difficult due to the heterogeneous nature of bio-signals.
- Modalities such as EEG, EOG, and ECG are highly susceptible to motion artifacts, especially during dizziness-provoked episodes.
- Dizziness-related physiological changes can be subtle and temporally dynamic, complicating accurate quantification.
- Early detection of dizziness before full symptom onset remains a major challenge.

This work presents a proof-of-concept engineering study. The system is evaluated in healthy participants performing controlled head-rotation tasks, and the term *dizziness levels* refers to a non-diagnostic, operational measure of motion-induced discomfort elicited during controlled stimulation and is not intended as a replacement for clinical assessment. Accordingly, EquilibriSense is positioned as a research platform for studying physiological responses to motion-induced dizziness under controlled conditions, rather than a system for direct vestibular function monitoring. Building on this framework, we make the following contributions.

- First, we introduce EquilibriSense, a head-mounted multimodal sensing platform for physiological characterization of motion-induced dizziness (see System Implementation Labeling in Results).
- Second, we propose a computational labeling framework to model the progression of dizziness under controlled stimulation (see EquilibriSense Motion-Induced Dizziness Quantification Framework in Methods).
- Third, we develop a dedicated, tailored algorithmic approach to classify dizziness levels (0–4) and early detect onset in real time (see Supplementary Modules SM 2–3).
- Fourth, we present an Advanced Data Conditioning Framework (ADCF), a preprocessing pipeline integrating the Stationary Wavelet Transform (SWT) and Artifact Subspace Reconstruction (ASR) for single-channel biosignal denoising (see Supplementary Module SM 1).
- Lastly, we evaluate the system in a controlled study involving 10 healthy participants, achieving 86.8% accuracy in multi-level classification and 98% accuracy in early onset detection.

Together, these contributions demonstrate the feasibility of multimodal physiological sensing and modeling of EquilibriSense for motion-induced dizziness under controlled conditions.

## Methods

EquilibriSense (Fig. 1) is a multimodal, head-mounted wearable platform designed to capture physiological responses associated with motion-induced dizziness under controlled experimental conditions. The system integrates multiple sensing modalities, including electroencephalography (EEG), electrooculography (EOG), electrocardiography (ECG), electrodermal activity (EDA), and inertial measurement unit (IMU) data. These signals are processed through a signal conditioning and feature extraction pipeline and analyzed using machine learning models to classify dizziness levels and detect onset.

### System implementation

The system implementation consists of hardware integration, signal acquisition, and preprocessing components designed to support multimodal physiological sensing in a wearable form factor (Fig. 1a,b). In particular, EquilibriSense is equipped with an array of physiological sensors and an OpenBCI control unit^23^ mounted on top of a standard 10–20 EEG cap. The lightweight, ergonomic cap ensures secure electrode contact for accurate brain activity measurement. Our key sensors are each strategically positioned to maximize classification accuracy while minimizing signal interference arising from motion artifacts, cross-channel coupling, and computational noise. Specifically, the EEG electrodes are distributed at O1, O2, C3, and C4 positions intentionally to support a lightweight wearable platform, prioritizing deployability over dense spatial coverage, and still provide comprehensive coverage for brain activity monitoring. The LIS3DH 3-axis accelerometer^24^ is built in the controller to track head motion with precision. We note that the EEG configuration provides limited spatial resolution and is not intended to infer activity from deep vestibular cortical regions such as PIVC or OP2, and IMU measurements reflect device motion rather than exact skull motion, and no external motion tracking validation was performed. Additionally, ECG electrodes are positioned on the mastoid processes to capture heart rate (HR) and heart rate variability (HRV), and the EDA module^25^ measures skin conductance responses via analog signals from the skin surface behind the ear. Among these sensors, EDA is sampled at 1 Hz to capture gradual changes in sweat to prevent cross-interference, while the remaining ones sample at 250 Hz to track high-resolution neural, cardiac, and motion dynamics.

Sensor data are then transmitted wirelessly via Bluetooth to a computer, where they undergo processing through a multi-stage pipeline (Fig. 1c) to mitigate artifacts and extract informative features, focusing on the ones that strongly correlate with dizziness-evoked physiological response patterns. These features are then input into various ML models to evaluate their effectiveness in categorizing five dizziness levels (0–4). The pipeline also includes a dizziness onset detection mechanism, enabling early intervention before symptoms fully manifest. We present all algorithms in detail in the Supplementary Materials.

### Study design

We recruited ten healthy adult participants (8 males and 2 females; age 18–50 years) with no self-reported history of vestibular disorders or chronic dizziness. Exclusion criteria included individuals over 50, minors, pregnant participants, and those with severe uncorrectable sensory impairments, neurological disorders, or skin conditions that could interfere with sensor placement. These criteria were implemented to reduce confounding factors such as age-related vestibular degeneration and to ensure consistent and secure sensor attachment across all subjects. The study protocol was reviewed and approved by the Institutional Review Board (IRB) of the University of Montana-Missoula (Protocol No. 16–24). All procedures were conducted in accordance with relevant guidelines and regulations. Informed consent was obtained from all subjects or their legal guardians prior to the study.

**Fig. 2:**
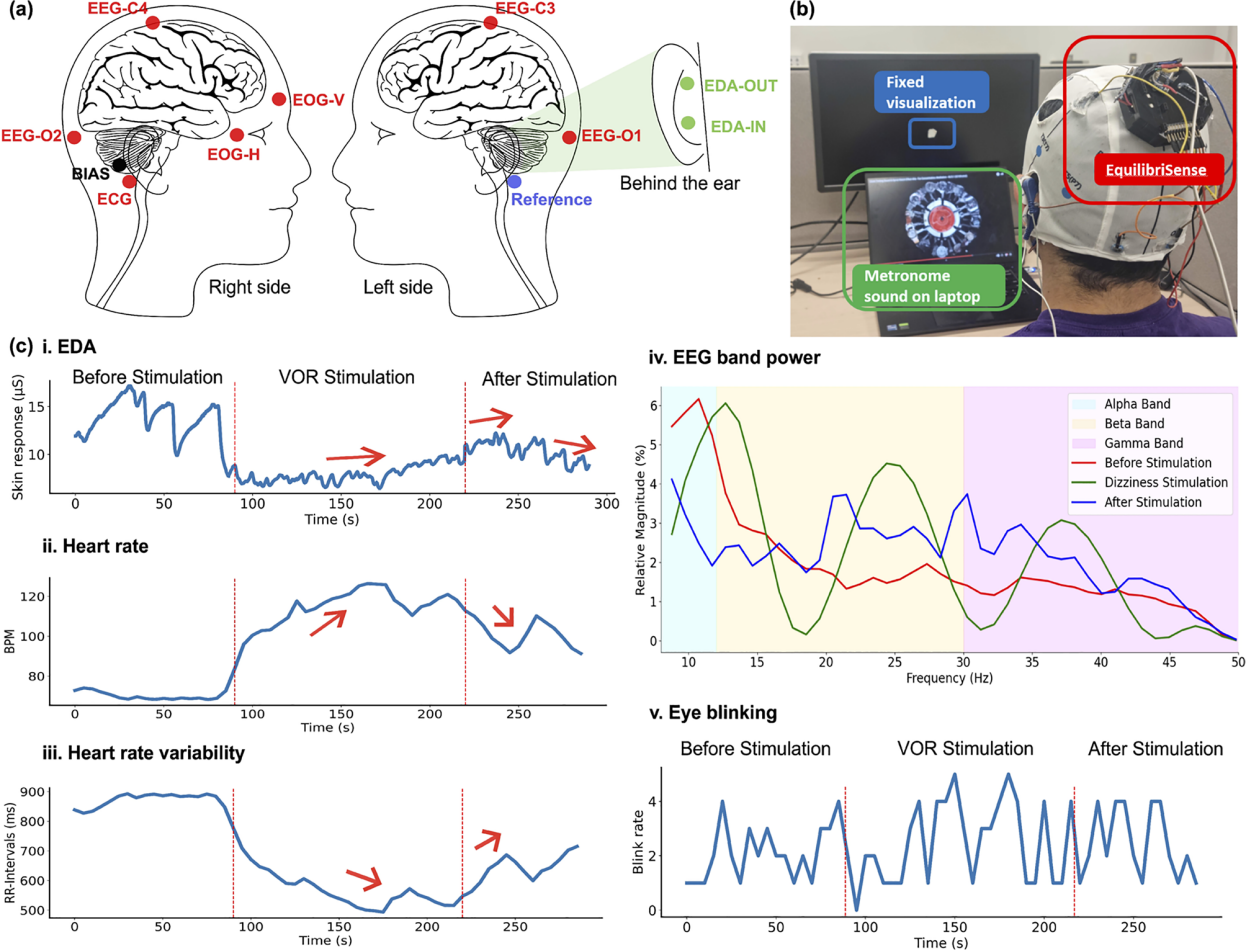
Electrode placement and physiological signal acquisition with EquilibriSense during dizziness-induced conditions. (**a**) Biopotential electrode placement on the left/right sides of the head and mastoid region. (**b**) Clinical experiment setup for inducing and monitoring dizziness. (**c**) Representative physiological responses, including skin conductance, cardiac activity (ECG), brain activity (EEG), and eye movements (EOG).

All participants completed demographic and medical history questionnaires, including the Dizziness Handicap Inventory (DHI)^26^, to confirm eligibility and establish the presence of any underlying dizziness impacting daily life. All experiments were conducted in a controlled laboratory setting. During data collection, participants were seated with the EquilibriSense system positioned on the head. Baseline physiological signals were recorded for 10 minutes in a relaxed, stationary condition to establish a reference state (Level 0). Dizziness was then induced using controlled head rotations along the yaw axis (left-right rotation) while maintaining an upright head posture and visual fixation on a static white dot on the monitor to promote consistent head-eye coordination (Fig. 2b). Head motion was guided by an auditory metronome with predefined frequencies (1–4 Hz). Prior to data collection, participants were familiarized with the protocol by listening to the metronome and performing practice head rotations to ensure consistent timing and coordination. Rotation amplitude and angular acceleration were not explicitly constrained but were performed within each participant’s comfortable range, with the requirement that the number of head rotations matched the metronome frequency, which was continuously recorded using the onboard IMU (see Supplementary Fig. SF5). Each participant completed four trials at rotation frequencies of 1, 2, 3, and 4 Hz, applied sequentially from low to high. Each trial lasted 2 minutes. Rest periods of 10 minutes (after 1 Hz and 2 Hz), 20 minutes (after 3 Hz), and 30 minutes (after 4 Hz) were provided between trials. These rest durations were determined empirically, and recovery was assessed based on participant-reported symptom resolution before proceeding to the next trial. All trials were conducted under supervision to ensure adherence to the prescribed motion protocol.

In this study, *dizziness* is operationally defined as motion-induced discomfort elicited during sustained head rotation under controlled conditions. This definition distinguishes dizziness from typical vestibular motion perception, which refers to the neutral awareness of self-motion during head movement and does not inherently involve discomfort or distress. Dizziness, as used here, is characterized by subjective unpleasant vestibular sensations (e.g., disorientation, instability, nausea) that arise beyond the expected sensation of motion itself during continued stimulation. This motion-based operational definition does not encompass non-motion-related symptoms such as light-headedness, presyncope, or spontaneous vertigo, nor does it imply clinical diagnosis of vestibular disorders. Accordingly, the proposed system does not classify motion perception itself, but rather quantifies physiological correlates associated with motion-induced discomfort.

To structure the experimental conditions, rotation frequencies were categorized into three groups, corresponding to increasing intensities of vestibular semi-circular canal stimulation. Low frequency (0 Hz, Level 0) resulted in negligible endolymph movement and minimal vestibular nerve activation, used to cause no motion-induced dizziness. Moderate frequencies (1–2 Hz; Levels 1–2) led to increased endolymph displacement and nerve firing rates, used to produce mild to moderate vestibular provoked dizziness. High frequencies (3–4 Hz; Levels 3–4) induced strong cupula deflections and elevated neural activity, used to produce overwhelming central compensation mechanisms and triggering more severe dizziness. These groupings were used to introduce controlled variations in motion intensity and do not represent clinically validated categories of vestibular response. The current framework also assumes a shared response model across participants and does not explicitly model inter-individual variability in dizziness sensitivity. Participants also reported perceived dizziness using a hand-sign scale from 0 (no dizziness) to 4 (extreme dizziness). Ratings were based on individual subjective perception and were not anchored to standardized symptom descriptors (e.g., nausea), nor were participants informed of the relationship between stimulation parameters and expected dizziness levels. These subjective ratings were collected for reference and comparison only and were not used as ground-truth labels for model training.

### EquilibriSense dizziness assessment framework

In this study, dizziness is treated as an operational construct defined within a controlled experimental paradigm. Rather than representing a clinical diagnosis, it corresponds to motion-induced discomfort elicited during active sustained sinusoidal head rotations. To enable real-time, objective dizziness quantification under controlled stimulation, we developed the EquilibriSense dizziness assessment framework, an integrated algorithmic pipeline designed to process multimodal physiological data acquired from the head-worn EquilibriSense device. This framework transforms raw biosignals into actionable insights regarding dizziness perception and onset, supporting continuous monitoring and early intervention in both clinical and real-world settings. The pipeline consists of three core computational modules:

1. An *Advanced Data Conditioning Framework* (ADCF) module (Supplementary Module SM 1), which performs real-time preprocessing and motion artifact removal on multimodal biosignals, including EEG, ECG, EOG, and IMU data, to ensure signal integrity and reliability in high-motion, noisy environments. It employs a hybrid ASR-IMU method with wavelet-based blind source separation to adaptively filter out artifacts at the single-channel level.
2. A *Physiological-based Dizziness Quantification Framework* module (Supplementary Module SM 2), which quantifies the body’s response to vestibular stimulation. Leveraging both time- and frequency-domain features across multiple sensor channels, this module extracts and selects discriminative features. Feature dimensionality is reduced through L1-based selection and Linear Discriminant Analysis (LDA), enabling high-accuracy, multi-level dizziness classification.
3. A *Dizziness Onset Detection* module (Supplementary Module SM 3), which employs a probabilistic ensemble model and a slope-based analysis of the dizziness likelihood curve to identify the early onset of dizziness. A Long Short-Term Memory (LSTM) neural network is further trained to anticipate the onset based on temporal trends, providing predictive capability prior to user awareness.

The entire pipeline operates with a median runtime of 0.77 seconds, enabling the inference of dizziness level from a 40-second data segment in near-real time. This performance validates the framework’s ability to support future closed-loop intervention in time-sensitive scenarios. The integration of high-fidelity physiological sensing, robust artifact mitigation, and neurocomputational modeling positions EquilibriSense as a powerful platform for quantifying dizziness and monitoring for provocation to vestibular stimulating movements, with applications in determining symptom severity and personalized neurorehabilitation.

### Objective labeling for dizziness quantification

Dizziness is a common symptom of the vestibular system’s response to intense stimulation and plays a central role in determining how a person responds to a provocative movement. To enable structured analysis of motion-induced dizziness, we propose a computational labeling framework that models the temporal evolution of discomfort during controlled active head rotation. The formulation is inspired by established descriptions of vestibular adaptation under sustained angular motion, in which perceived intensity rises and gradually stabilizes over time. Rather than serving as a direct physiological measurement, this model provides a principled and repeatable way to generate labels under controlled experimental conditions.

This framework is mathematically described using a first-order exponential model^27^, motivated by the adaptation behavior of the semicircular canals under sustained active angular motion^28^. Specifically, the dizziness level $R(t)$ is modeled as a function of time and rotation frequency, given by:

1 $$\begin{aligned} R(t) = D_{\text {max}} \times \left( 1 - e^{-\frac{t}{\tau }} \right) , \end{aligned}$$

where $D_{\text {max}}$ represents the maximum dizziness level associated with a given rotation frequency, $t$ denotes elapsed time, and $\tau$ is the vestibular system’s time constant governing the rate of increase, typically ranging from 5 to 15 seconds^29^. Based on established physiological studies^30^, we adopt $\tau = 6 \, \text {seconds}$ as a representative value. To capture the nonlinear increase in vestibular stimulation with higher frequencies, $D_{\text {max}}$ is scaled with rotation frequency (1–4 Hz) to introduce controlled variation in motion intensity. The model’s predictions align with known vestibular behavior and prior empirical research^31^, capturing the characteristic rise and plateau of dizziness levels under continuous head rotation. As shown in Fig. 5a, higher frequencies induce faster and more intense dizziness responses, validating the frequency-dependent scaling of $D_{\text {max}}$.

This physiological framework offers several advantages over traditional linear labeling schemes. By modeling dizziness as a smooth, continuous process governed by neurophysiological principles, it provides more realistic and interpretable labels. The use of floating-point labels enhances label granularity and allows for finer resolution in dizziness quantification. For integration into classification tasks, these values can be discretized (e.g., 2.4 $\rightarrow$ 2, 2.6 $\rightarrow$ 3) without loss of fidelity. Moreover, the exponential formulation ensures smooth transitions in dizziness levels, avoiding abrupt or artificial changes. Therefore, this labeling framework serves as a structured computational proxy derived from stimulation parameters (time and rotation frequency) and does not represent a clinical ground truth of dizziness induction. The resulting labels provide a consistent supervisory signal for machine learning within the experimental paradigm, rather than a direct measure of subjective experience.

Machine learning models are trained exclusively on physiological signals (EEG, EOG, ECG, EDA, IMU-derived features) without direct access to stimulation parameters. However, because physiological responses may correlate with motion intensity, the learned representations may partially encode frequency-related patterns. This limitation should be considered when interpreting classification performance.

## Results

This section presents the performance of EquilibriSense under the controlled laboratory conditions, and all results should be interpreted within this constrained experimental setup described in Methods. We first evaluate signal quality and acquisition reliability, followed by classification and onset detection performance.

### Assessment of reliable bio-signal acquisition under vestibular stimulation

In this section, we evaluate the design and signal characteristics of EquilibriSense, detailing the sensor layout (Fig. 2a), choice of electrode materials, and integration of sensing components, under vestibular stimulation (Fig. 2b). The dizziness-induced protocol is described in more detail in Section Methods.

Specifically, to optimize form factor and signal quality in a head-mounted design, we carefully selected sensor placements. For EEG, we focus on monitoring changes in Alpha, Beta, and Gamma power spectral density bands, key indicators of neural responses to vestibular stimuli^32^. To achieve this, four electrode positions (O1, O2, C3, and C4) were chosen to provide broad cortical coverage while minimizing hardware complexity. However, capturing EEG signals on the scalp can be challenging due to the presence of hair^33^. To address this, dry comb electrodes are used for EEG to improve contact through hair. We also record ECG at the mastoid, offering consistent skin contact and reduced susceptibility to specific motion artifacts (e.g., movement-induced electrode-skin impedance fluctuations and cable motion artifacts), primarily due to improved electrode fixation and mechanical stability over a rigid bony surface^34^. EOG configuration is simplified by omitting two standard electrodes: one on the left eye (horizontal EOG) and one below the right eye (vertical EOG), preserving eye movement tracking while reducing footprint. The EDA sensor is positioned slightly behind the ear on the helix to mitigate interference with other sensors due to its measuring mechanism, which injects a small current into the skin and measures resulting voltage changes, enabling detection of skin conductance fluctuations. Placement on the ear helix also avoids interference from muscle or bone conductivity that might conduct electricity. Wet disposable electrodes are applied for EOG, ECG, EDA, and EEG reference channels to enhance skin contact and minimize motion artifacts. Finally, the IMU sensor is placed at the top of the head to comprehensively capture head movement dynamics. We note that scalp-mounted sensors may experience relative motion between the scalp and skull, particularly when additional device mass is present. In the current system, such relative motion is not independently measured or explicitly modeled, but is treated as a source of motion-related noise.

Fig. 2c illustrates signal patterns captured under a controlled vestibular stimulation protocol, validating the sensor configuration and system responsiveness.

### Motion artifacts removal

To evaluate the effectiveness of the proposed motion artifact removal technique (ADCF), detailed in Supplementary Module SM1, we primarily use metrics such as signal-to-noise ratio (SNR), root mean square error (RMSE), and classification accuracy of physiological events^35^. However, most existing evaluations rely on relative comparisons between methods without an absolute ground truth, limiting the reliability of performance assessments. Establishing a standardized ground truth protocol is critical for validating artifact removal approaches against a consistent reference.

**Fig. 3:**
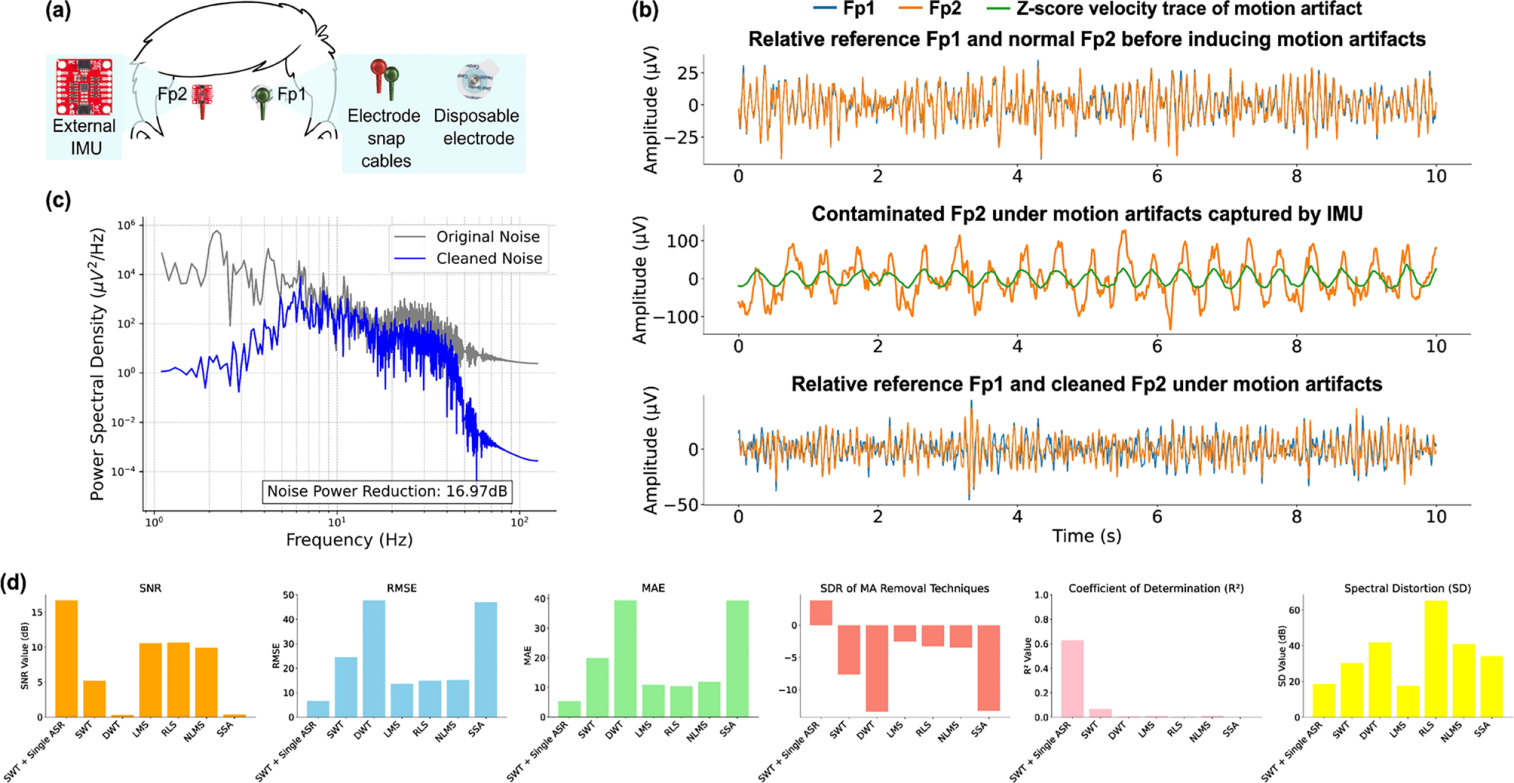
Performance of the Advanced Data Conditioning Framework (ADCF) for motion artifact removal. (**a**) Ground truth setup using dual-electrode configuration. (**b**) Comparison of ground truth, contaminated, and cleaned signals after artifact removal. (**c**) Analysis of noise reduction effectiveness. (**d**) Quantitative comparison of performance metrics across various motion artifact removal techniques.

As demonstrated in Fig. 3a, we developed a validation method using two wet electrodes positioned on the forehead: one (Fp1) serving as a relative reference channel, and the other (Fp2) as the artifact-contaminated channel. In this configuration, an external IMU sensor was attached to the snap cable of the Fp2 electrode to capture motion patterns, while motion artifacts were manually induced by pressing and scrolling on the electrode. Since Fp1 and Fp2 are spatially distinct, their recordings may differ due to local cortical contributions and electrode-skin impedance, and thus Fp1 should not be interpreted as an absolute ground-truth signal. Rather, both channels share a common baseline in that they reflect the subject’s underlying frontal EEG activity recorded under the same acquisition system, while Fp2 is additionally subjected to controlled mechanical perturbations to induce motion artifacts. We note that physically perturbing one electrode may introduce correlated artifacts in nearby channels due to scalp deformation, cable coupling, and common-mode effects. To strengthen confidence, future validation will include tests with simulated or semi-simulated signals in which known clean signals are combined with controlled artifact waveforms, enabling evaluation against a ground truth known by construction.

In Fig. 3b, motion artifacts are clearly evident in the contaminated Fp2 EEG signal, correlating strongly with the z-score velocity trace from the IMU. After applying our artifact removal pipeline, which integrates IMU data with Stationary Wavelet Transform (SWT) and Artifact Subspace Reconstruction (ASR), the cleaned Fp2 signal closely aligns with the Fp1 reference, demonstrating effective noise reduction. Additionally, power spectral density analysis (Fig. 3c) further confirms this, showing a 16.97 dB reduction in noise power across the frequency spectrum.

Comparative results in Fig. 3d show our proposed approach (i.e., an integration of IMU, SWT, and ASR) outperforms common artifact removal methods across multiple metrics during head rotation. It achieves the highest SNR (>16 dB), lowest RMSE ($\sim$6), and lowest Mean Absolute Error (MAE, $\sim$5). Additionally, it records the highest Signal Distortion Ratio (SDR = 4), the highest Coefficient of Determination (R$^2$ = 0.6), and the lowest Spectral Distortion (SD < 20 dB). These results indicate superior noise suppression performance and enhanced fidelity in preserving the underlying physiological signals.

Collectively, these findings validate the efficacy of our artifact removal strategy and underscore the importance of using a controlled ground truth protocol in performance evaluation.

### Dizziness level classification performance

To evaluate both the original 5-class dizziness scale and address labeling ambiguity, particularly between adjacent levels such as 1 and 2 or 3 and 4, we assessed the performance of dizziness quantification pipeline, detailed in Supplementary Module SM2, across three labeling schemes: (1) the original 5-class configuration, (2) a 4-class version with Levels 1 and 2 merged, and (3) a 3-class version with both Levels 1–2 and 3–4 combined. Note that the 5-level scale is our intentional design choice to capture finer gradations of motion-induced vestibular discomfort, while the 3- and 4-class configurations are evaluated to examine robustness under coarser labeling.

**Fig. 4:**
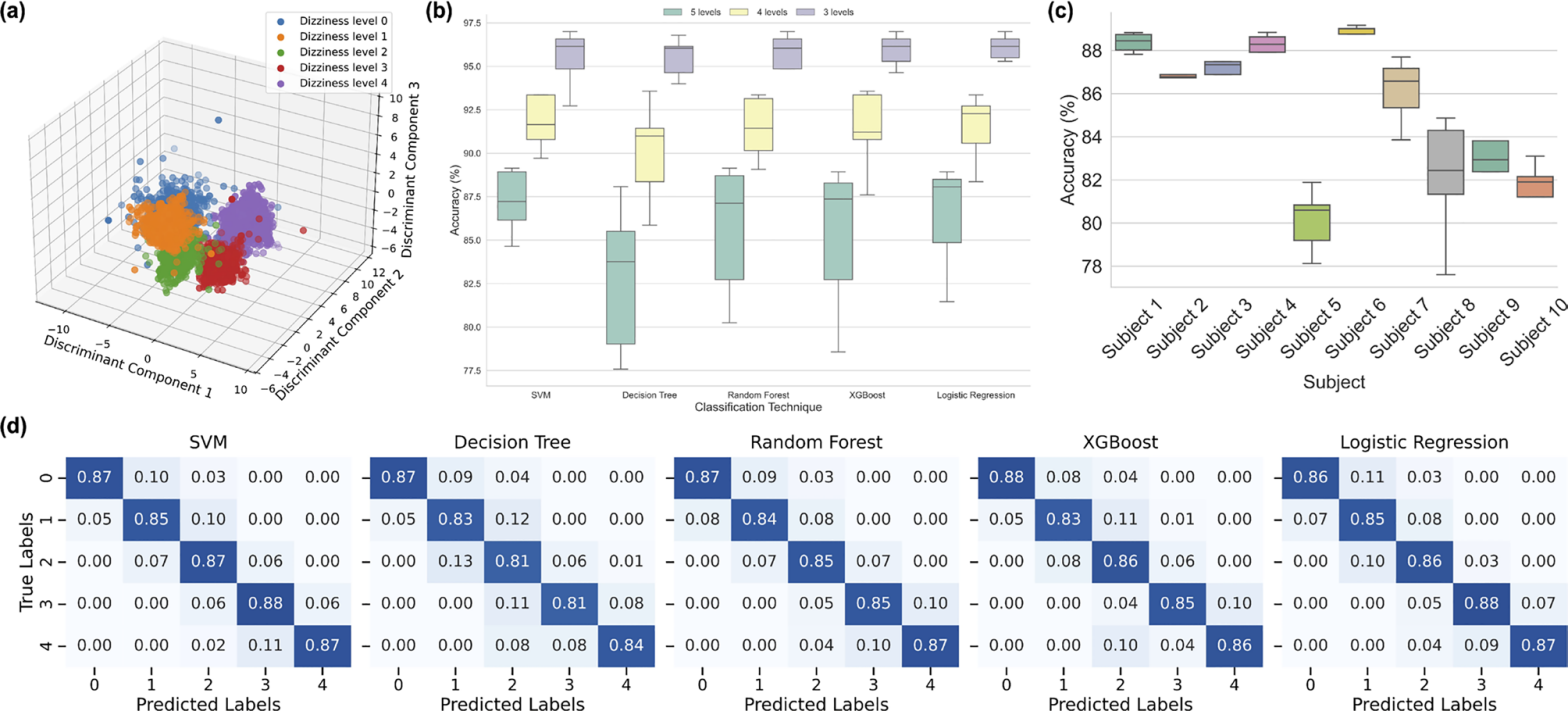
Accuracy performance of the EquilibriSense dizziness quantification model in the original 5-level scale. (**a**) Three-dimensional projection of feature representations using LDA. The visualization illustrates relative separability between classes in the projected space and does not represent classifier decision boundaries. Distances and separations in this plot should be interpreted qualitatively. (**b**) Average classification accuracy distribution across different machine learning models and labeling schemes. (**c**) Average accuracy distribution across individual subjects, highlighting inter-subject variability. (**d**) Confusion matrix illustrating model performance in classifying five dizziness levels.

Feature selection was performed using L1-regularized logistic regression on the initial 121-feature set, yielding 80, 77, and 71 features for the 5-, 4-, and 3-class datasets, respectively. This step is critical for enhancing downstream classification performance in machine learning models. These selected features were further reduced using Linear Discriminant Analysis (LDA), with the dimensionality equal to the number of classes minus one. To visualize class separability, we plotted the top LDA components in three dimensions for the 5- and 4-class datasets, and in two dimensions for the 3-class dataset (Fig. 4a and Supplementary Figs. SF1 and SF2). The projected feature space shows observable clustering trends across dizziness levels, suggesting that the extracted features capture structured differences between conditions. These projections are intended for visualization purposes only and do not reflect the actual decision boundaries learned by the classification models. We note that dimensionality reduction techniques such as LDA provide a simplified view of high-dimensional feature spaces. As such, apparent overlaps or separations in the projected space may not directly correspond to classification performance in the original feature space. As shown in these figures, the resulting plots reveal that class clusters become more distinct as the number of dizziness levels is reduced. Specifically, the data points are more cohesively grouped and better separated in the 3-class configuration compared to the 5-class version. This progressive improvement in cluster separation suggests enhanced discriminability of the model under coarser labeling schemes, potentially leading to more robust and accurate classification performance. These qualitative observations are supported by the quantitative classification results presented below.

We evaluated five machine learning models, including SVM, Decision Tree, Random Forest, XGBoost, and Logistic Regression, for motion-induced dizziness quantification using Leave-One-Out Cross-Validation (LOOCV) performed at the subject level across the 10 participants. In each iteration, data from one participant were held out for testing, while models were trained on the remaining participants, ensuring comprehensive, subject-independent evaluation. Minority class weights were adjusted during training to counteract imbalance and improve classification fairness. Performance metrics, including accuracy, precision, and recall, were averaged across all LOOCV iterations to obtain robust model evaluations. For the 5-class scenario (Fig. 4d), Random Forest and XGBoost showed robustness with class accuracies between 0.84 and 0.87, while Decision Tree and Logistic Regression showed greater variability, particularly in the lower classes. On the other hand, for the 3-class scenario (Supplementary Figs. SF3), all models achieved high true positive rates. Random Forest and XGBoost performed best, with accuracies of 0.95 - 0.96 across classes. Logistic Regression followed closely. In the 4-class setup (Supplementary Figs. SF4), SVM and XGBoost maintained a strong balance between precision and recall, while Random Forest and Logistic Regression retained class-wise accuracies above 0.91, except for a slight drop in the most challenging class (e.g., 0.89 in Random Forest for the 4th class).

The accuracy distribution across all models and labeling schemes is presented in Fig. 4b. In summary, XGBoost achieved the highest median accuracy (96%) in the 3-class configuration with minimal variance, followed closely by Random Forest and Logistic Regression. For 4-class labeling, model accuracies remained above 90% with reduced variance. The more complex 5-class case showed increased variability, though SVM maintained the highest median performance ($\sim$87%). Although the 3-class configuration demonstrated improved class separability and reduced variance, the multi-level (5-class) scale provides higher-resolution insight into transitional vestibular responses and was therefore retained for comprehensive analysis. In Fig. 4c, the box plot illustrates the variability in accuracy across subjects. This subject-level analysis showed consistent performance for some subjects (e.g., Subjects 4 and 10), while others (e.g., Subjects 5 and 8) exhibited greater accuracy spread, indicating inter-subject variability in physiological responses.

Because the labeling framework is parameterized by rotation frequency and time, and physiological responses may correlate with these factors, classification performance may partially reflect frequency-related information rather than purely intrinsic physiological responses.

### Objective labeling

To address class imbalance in the dataset, particularly the dominance of Class 0 (no dizziness), we applied different segmentation strategies during labeling. For baseline data (10 minutes) with stable physiological patterns, a 10% overlap between windows was sufficient. In contrast, data collected during and immediately after vestibular stimulation (up to 2 minutes) were segmented with 90% overlap to better capture transient dizziness responses and increase the representation of symptomatic classes.

**Fig. 5:**
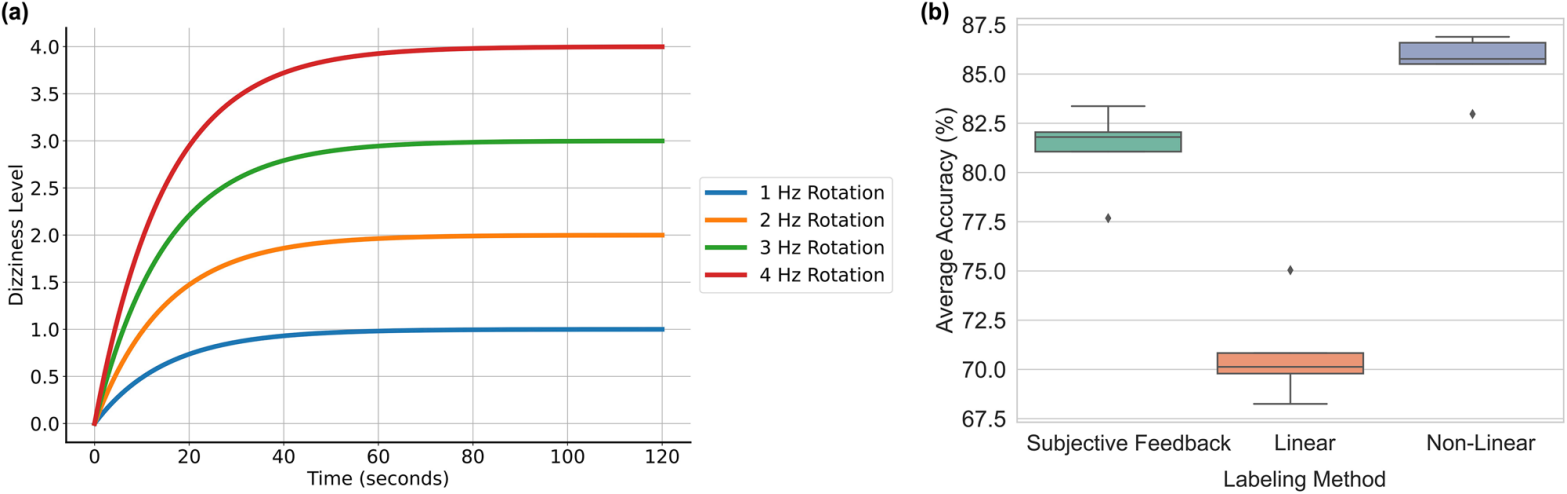
Accuracy performance of objective labeling. (**a**) Modeled changes in dizziness level over time across different head rotation frequencies using an exponential-based physiological framework. (**b**) Comparison of average classification accuracy across three labeling methods: subjective feedback, linear interpolation, and non-linear (objective) labeling.

In addition to collecting subjective dizziness ratings from participants using a 0–4 hand-sign scale (0: no dizziness; 4: extreme dizziness), we employed a computational labeling framework to quantify the temporal progression of motion-induced dizziness during head rotations. As described in the Methods, these labels are derived from a model parameterized by stimulation conditions and should be interpreted as structured proxies rather than direct measures of vestibular function. All machine learning models were trained exclusively using these computed labels, while subjective ratings were used only as an independent reference for qualitative comparison. We note that, because the labeling framework is linked to stimulation parameters, and physiological responses may correlate with these parameters, the learned representations may partially reflect frequency-related patterns. This formulation prioritizes consistency and reproducibility within the controlled experimental paradigm.

Figure 5a shows how predicted dizziness levels evolve over time at different rotation frequencies, with higher frequencies eliciting faster and more intense responses. The modeled curve, characterized by a non-linear rise and plateau, reflects the vestibular system’s dynamic adaptation to sustained head rotations. Figure 5b compares the average accuracy of dizziness quantification across three labeling methods: subjective feedback, linear interpolation, and our proposed non-linear physiological model. As shown in the figure, labels derived from the physiological model exhibit closer alignment with observed physiological response patterns than linear interpolation or subjective labeling alone, supporting their use as a consistent supervisory signal for training. In particular, the non-linear method outperforms the others, achieving the highest median accuracy of approximately 85.77% with minimal variance, as evidenced by a narrow interquartile range. In contrast, subjective labeling yields a moderate accuracy of around 82% with slightly higher variability and some outliers, highlighting inconsistent alignment with physiological responses across trials. It is important to note that subjective labeling might depend heavily on the individual subject’s perception and self-assessment, which introduces variability and potential bias in the labeling process. The linear labeling method performs worst, with a median accuracy of $\sim$70%, likely due to its oversimplified representation of dizziness progression.

These results emphasize the importance of aligning data labeling with underlying physiological principles. The non-linear (exponential-based) labeling approach offers a more accurate and reliable foundation for dizziness quantification, enhancing both model performance and interpretability.

### Dizziness onset detection

Anticipating dizziness onset before it becomes perceptible is a critical feature of our system, enabling timely intervention and risk mitigation. We define dizziness onset as the initial phase when symptoms begin to emerge but are not yet consciously perceived, a point that varies across individuals, making it difficult to identify using subjective feedback alone.

**Fig. 6:**
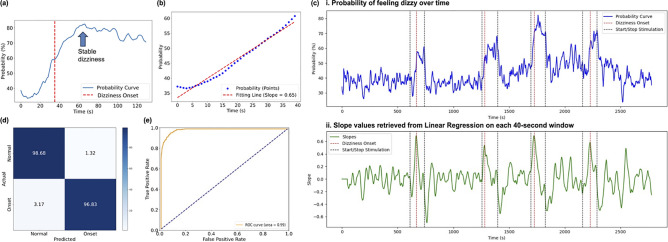
Dizziness onset detection performance. (**a**) Temporal change in dizziness probability during vestibular stimulation. (**b**) Slope values derived from Linear Regression fitting within a sliding window. (**c**) Synchronization between the probability curve and slope values over time. (**d**) Confusion matrix showing classification performance for normal vs. onset states. (**e**) ROC curve for dizziness onset detection.

Figure 6a illustrates the progression of dizziness probability over time during vestibular stimulation, rising from below 40% to over 80%, then fluctuating around 75%. This pattern reflects the transition from pre-onset (i.e., a low likelihood of dizziness) to a full experience. Detecting onset involves identifying this transition before the probability stabilizes. Our detection framework, detailed in Supplementary Module SM3, first employs linear regression to assess the slope of probability increase within a 40-second window (Fig. 6b). As shown in Fig. 6c, the steepest slope, marked on the rising edge of the probability curve, captures the earliest indication of dizziness onset (i.e., the moment when dizziness begins to emerge but has not yet stabilized), providing a valuable lead time for intervention.

To automate onset detection, we trained a Long Short-Term Memory (LSTM) model to classify normal vs. onset states. The resulting confusion matrix (Fig. 6d) shows high classification accuracy: 98.68% for normal and 96.83% for onset instances, minimizing both false positives (1.32%) and false negatives (3.17%). The model’s precision and recall remain consistently above 94%, with an F1 score reflecting well-balanced performance in identifying both normal and onset states. On the other hand, the distribution of evaluation metrics provides additional insight into the model’s performance. The accuracy of the model is consistently high, with a median value exceeding 98%. The ROC curve (Fig. 6e) demonstrates the model’s ability to achieve excellent separability between the two classes, with an area under the curve (AUC) of 0.99, indicating high sensitivity and specificity.

We further analyzed the LSTM model’s robustness across different train-test splits (Supplementary Table ST1). Accuracy ranged from 93% to 95%, with precision consistently at 1.00 in most configurations. Recall varied from 0.77 to 0.93, reflecting variation in true positive rates across datasets. F1 scores remained stable between 0.87 and 0.92, confirming balanced detection performance regardless of data partitioning.

These results validate the feasibility and reliability of real-time dizziness onset detection using physiological trends, offering a proactive mechanism for early intervention in vestibular-related conditions.

### Ablation study on model performance

Ablation experiments were conducted to assess the individual contributions of sensor modalities and preprocessing steps to overall model performance.

**Fig. 7:**
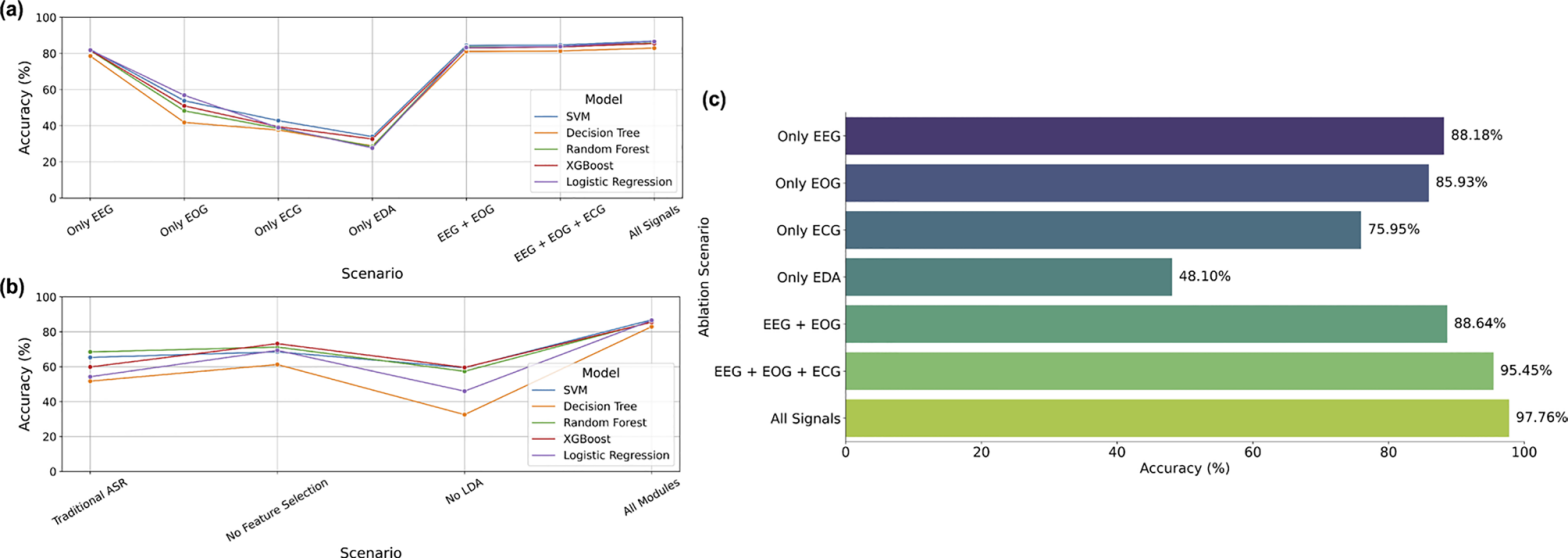
Ablation analysis. (**a**) Classification accuracies of five models under different sensor modality ablation conditions. (**b**) Classification accuracies of five models under ablation of feature selection and preprocessing components. (**c**) Classification accuracies for dizziness onset detection across various sensor modality ablation scenarios.

As shown in Fig. 7a, EEG provides strong task-related signals under the experimental paradigm, reflecting a combination of neural and motion-related (i.e., visual, motor, and oculomotor) processes. Removing EEG data caused model accuracies to drop below 70% across all classifiers, highlighting the central role of brain activity in capturing dizziness-related patterns. In contrast, excluding EOG, ECG, or EDA resulted in more modest performance declines, with accuracies remaining around 80%, indicating that these modalities serve as complementary sources of information.

Figure 7b illustrates the importance of preprocessing and feature engineering. Eliminating L1-based feature selection led to noticeable accuracy reductions, while the exclusion of LDA produced the most substantial performance degradation, bringing accuracies below 60% for most models. These results underscore the necessity of both feature selection and dimensionality reduction for achieving optimal classification performance.

Finally, in Fig. 7c, we evaluate dizziness onset detection under various sensor combinations. Using all modalities yields the highest classification accuracy (97.76%). EEG alone achieves 88.18%, while combining EEG with EOG and ECG boosts accuracy to 95.45%, demonstrating their synergistic nature in identifying dizziness onset. Conversely, EDA alone results in the lowest accuracy (48.10%), indicating limited standalone predictive utility for onset detection.

### Real-time performance

**Fig. 8:**
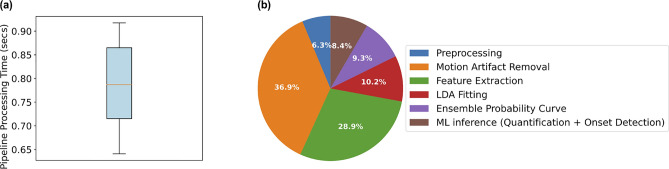
Real-time processing performance. (**a**) Processing time per cycle across the full signal analysis pipeline. (**b**) Runtime distribution across individual pipeline components, highlighting relative computational load.

EquilibriSense exhibits consistent and efficient real-time processing performance, with a median processing time of approximately 0.77 seconds per cycle (Fig. 8a). The boxplot shows a range of processing times spanning from approximately 0.64 seconds to 0.92 seconds, reflecting the pipeline’s stability under various operating conditions. The system employs a 1-second stride for windowed signal processing and machine learning inference, ensuring responsiveness suitable for continuous monitoring applications. This configuration strikes a balance between computational efficiency and detection accuracy, supporting timely feedback for dizziness monitoring and early intervention. Processing times remain well below the 1-second threshold, confirming the system’s real-time capability. As shown in Fig. 8b, runtime distribution analysis reveals the relative computational load across different pipeline tasks, with motion artifact (MA) removal accounting for the largest computational load (36.9%), followed by feature extraction and selection.

For power consumption assessment, we monitored the current draw of the EquilibriSense device. Under full-feature operation, the system consistently consumed approximately 51.2 mA. During stress testing, charging a 1000 mAh battery to full capacity and discharging it to depletion, the system operated for nearly 16 hours. While the theoretical battery life is approximately 19.5 hours for a 1000 mAh battery, the observed duration was slightly reduced, likely due to battery aging and transient current spikes during active processing.

To improve energy efficiency, several hardware-level optimizations were applied. The MCU clock speed on the PIC32 ChipKIT was reduced from 48 MHz to 12 MHz, maintaining data throughput while lowering power draw. Additionally, the unused 8th channel of the ADS1299 was disabled to conserve energy. While reducing the analog front-end (AFE) amplifier gain from 24 to 12 could have saved an additional 6 mA, this option was not pursued to preserve signal fidelity. Post-optimization, current consumption decreased to approximately 43.1 mA, extending battery life by an estimated 4 hours for a 1000 mAh battery and nearly 12 hours for a 500 mAh battery. These optimizations significantly enhance power efficiency without sacrificing system performance, supporting long-duration operation in real-world, continuous monitoring scenarios.

## Discussion

Our paper presents EquilibriSense, a multimodal wearable system designed for quantifying motion-induced dizziness under controlled experimental conditions. Through the integration of diverse physiological sensors and advanced signal processing pipelines, the system demonstrates the feasibility of classifying dizziness levels and detecting onset in a structured active head-rotation paradigm. While the results highlight the potential of multimodal physiological sensing for characterizing motion-induced discomfort, several limitations and opportunities for improvement remain.

*Scope and Positioning:* EquilibriSense is designed as a proof-of-concept research platform rather than a clinical diagnostic tool. The system is evaluated exclusively in healthy participants under controlled stimulation, and the defined dizziness levels represent a non-diagnostic, operational measure of motion-induced discomfort. Accordingly, the system has not been validated against gold-standard clinical assessments such as videonystagmography (VNG), video head impulse testing (vHIT), or rotational chair testing. The current findings, therefore, demonstrate feasibility within a controlled experimental setting and should not be interpreted as direct measures of vestibular function.

*Hardware Design and Mobility Limitations:* The current implementation of EquilibriSense, featuring the OpenBCI board mounted atop a 10–20 EEG electrode cap, presents notable constraints on user mobility and comfort. The rigid form factor and relatively bulky control circuitry, while effective in laboratory settings, limit usability in real-world scenarios involving movement. This design restricts practical applications to minimally dynamic environments, such as seated assessments or controlled clinical tasks. To enhance wearability and broaden use cases, future iterations should prioritize integration into more ergonomic and flexible platforms, such as smart headbands, augmented-reality eyeglasses, or even everyday apparel like caps or visors. These design advancements would not only improve user comfort and mobility but also expand the system’s adoption in ambulatory monitoring, workplace ergonomics, and extended daily wear.

*Validation of Head Motion Measurements:* Angular velocity profiles and precise kinematic properties were not explicitly quantified beyond frequency control. In addition, the similarity between motion captured by the device-mounted IMU and true head or skull motion was not explicitly quantified in this study. Direct validation using external motion references (e.g., optical tracking or bone-anchored sensors) represents an important direction for future work, particularly for improving biomechanical fidelity in head-worn sensing systems.

*Sensitivity of the IMU Sensor:* The system currently employs the LIS3DH 3-axis accelerometer, which, while sufficient for capturing gross head movements, may lack the sensitivity and resolution required to detect subtle micro-movements critical for vestibular assessments. Such fine-grained motion data are often necessary to detect early signs of dizziness and to disambiguate signal artifacts from legitimate physiological events. This limitation highlights the need to integrate higher-performance IMUs that offer lower noise floors, higher sampling rates, and finer resolution. Incorporating next-generation IMUs would significantly improve the system’s fidelity in detecting nuanced motion-induced dizziness-evoked physiological changes and enhance overall signal interpretability.

*Physiological Interpretation and Labeling Limitations:* The proposed labeling approach is based on a computational model derived from controlled stimulation parameters and serves as a structured experimental proxy rather than a clinical ground truth. Although machine learning models are trained solely on physiological signals, these signals may correlate with stimulation conditions, and thus the learned representations may partially encode stimulus-related patterns. Furthermore, physiological signals recorded during active head motion may reflect a mixture of processes, including neural, oculomotor, autonomic, and motion-related artifacts. To partially address this, we analyze post-stimulation periods, where head motion ceases, and observe persistent distinguishable patterns, suggesting that the system captures sustained physiological correlates of dizziness beyond immediate motion effects. However, isolating vestibular-specific contributions remains an open challenge.

*Environmental Generalizability:* Although EquilibriSense has demonstrated strong performance in controlled laboratory conditions, its generalizability to real-world environments remains to be validated. The current system has been optimized under conditions with limited ambient variability, such as stable lighting, minimal motion interference, and controlled noise levels. For practical deployment, however, the system must reliably perform during a broad spectrum of daily activities, such as walking, cooking, driving, or exercising. Future work should include rigorous field testing across diverse and dynamic real-life scenarios (e.g., sports) to evaluate robustness, validate model generalization, and identify any failure points or environmental sensitivities that may hinder performance.

*Protocol and Labeling Consistency:* One observed challenge during experimental trials was the inconsistency in how participants transitioned between different levels of induced dizziness. This variability is likely due to individual physiological differences or slight deviations in adherence to the stimulation protocol. Such inconsistencies can complicate labeling accuracy and reduce the clarity of physiological signal interpretation. Addressing this issue will require stricter protocol enforcement, enhanced participant training, and potentially the development of automated dizziness induction mechanisms to ensure more consistent and reproducible labeling. These improvements will contribute to more reliable labeling and better model training.

*Dataset Size and Diversity:* The study is based on a relatively small cohort of healthy individuals (n = 10), which limits statistical power and generalizability to pathological vestibular conditions. In addition, the current framework assumes a shared response model across participants and does not explicitly account for inter-individual variability in dizziness sensitivity or adaptation. Expanding the dataset to include larger and more diverse populations, including clinical cohorts, will be essential for evaluating robustness and improving model generalization.

*Feature Representation and Generalizability:* Although this study employs controlled active sinusoidal head rotation as a repeatable stimulus to elicit motion-induced dizziness, the primary insight lies in identifying the *types* of EEG feature representations that encode dizziness-related cortical responses, rather than the specific quantitative expressions of those features under a particular stimulus. Feature categories such as spectral power modulation, band-power ratios, and non-linear complexity measures consistently contributed to classification performance, indicating that these representations capture broader neural processes associated with vestibular processing and sensory conflict. While the magnitude and frequency-specific expression of these features (e.g., within specific Alpha or Beta bands) may vary across different dizziness-inducing conditions, their relevance as informative feature classes is not inherently tied to sinusoidal motion. In this work, sinusoidal head rotation serves as a systematic and repeatable stimulus to validate the development of a lightweight, multimodal sensing and analysis framework for quantifying dizziness-related physiological responses. Evaluating the extent to which these feature representations generalize across different motion conditions or real-world scenarios remains to be validated.

## Supplementary Information

Below is the link to the electronic supplementary material.

Supplementary Information.

## Data Availability

The datasets generated and/or analyzed during the current study are available from the corresponding author upon reasonable request for research purposes, subject to institutional and ethical approval.

## Code availability

The code used for signal processing, feature extraction, and machine learning analysis is publicly available at DOI: 10.5281/zenodo.18158416 via Zenodo^36^. The repository includes version-controlled code, documentation, and instructions for reproducing the results. The code is released under an open-source license (Apache License 2.0) compliant with Open Source Initiative guidelines.
