# Supplementary material for: Objective quantification of motion-induced dizziness using a proof-of-concept multimodal wearable platform
Source: Sci Rep. 2026 May 11;16:21543. doi: 10.1038/s41598-026-52443-5 (PMC13350944; doi:10.1038/s41598-026-52443-5)
Supplement: Supplementary file 1 — Supplementary Information. [file 41598_2026_52443_MOESM1_ESM.pdf]

Supplementary Information for Objective Quantification of Motion-Induced Dizziness Using a Proof-of-Concept Multimodal Wearable Platform

Nhan Cao[1], Brian Loyd[2], Andy Kittelson[2], and Anh Nguyen[1,*]

[1]University of Montana, Department of Computer Science, Missoula, MT 59812, USA
[2]University of Montana, School of Physical Therapy and Rehabilitation Science, Missoula, MT 59812, USA
[*]anh.nguyen@umontana.edu

Supplementary Information

Supplementary Module SM 1: Advanced Data Conditioning Framework (ADCF) for Real-Time Physiological Signal Cleaning

The Advanced Data Conditioning Framework (ADCF) is designed to enhance the integrity and reliability of physiological signals collected by the head-worn EquilibriSense system. It focuses on three primary functions: signal preprocessing, real-time motion detection, and artifact removal, particularly for dynamic environments involving head movements.

Signal Preprocessing

To prepare physiological signals for analysis, we first apply a bandpass filter (1–50 Hz) to EEG, ECG, and EOG channels, preserving frequency bands relevant to neurophysiological activity while removing low-frequency drift and high-frequency noise. A notch filter at 50/60 Hz is also used to suppress powerline interference. IMU signals are bandpass-filtered from 1–20 Hz to isolate motion-relevant frequencies. The EDA signal, being low-frequency and containing meaningful DC trends, is left unfiltered to preserve the underlying physiological variation.

Motion Detection Strategy

To identify motion-contaminated segments, we calculate the vector sum of the tri-axial acceleration data over 10-second windows. Each window is further divided into overlapping 2-second slices (50% overlap). The average energy within each slice is compared against a threshold derived from static-condition calibration data. Windows containing at least one slice above this threshold are marked as contaminated and flagged for artifact removal.

Motion Artifact Management

Accurate removal of motion artifacts is essential for preserving the diagnostic value of signals like EEG, ECG, and EOG[1]. The artifacts, often caused by head movements or other body activities[2], can distort the physiological signals, leading to misinterpretations. Traditional techniques, such as Independent Component Analysis (ICA)[3,4], Canonical Correlation Analysis (CCA)[5,6], Adaptive Filtering[7,8], and Wavelet Transform[9,10], face limitations in real-time applications due to high computational demand, static assumptions, or poor adaptability to dynamic noise profiles. Artifact Subspace Reconstruction (ASR)[11,12] has emerged as a preferred method for real-time cleaning due to its speed and ability to reject non-stationary noise without heavy computation.

The ASR framework[13] operates on the principle that a data slice, D_t, can be decomposed into its components, C_t, using a transformation matrix, T_r:

$$D_t = T_r C_t \tag{1}$$

Due to[14], artifact removal is executed within the principal component (PC), expressed as $P_t = U_t^T D_t = U_t^T T_r C_t$. This facilitates the isolation and extraction of artifacts. The clean components, $(C_t)_{\text{clean}}$, are then reconstructed using the pseudoinverse of the truncated matrix $U_t^T T_r$.

ASR applies an eigenvalue decomposition to the covariance matrix taken across channels of the IIR-filtered uncleaned bio signal segments $Cov(D_t) = U_t E_t U_t^T$ along a preconfigured sliding window. For each window, ASR checks if the variance of the jth principal component $(U_t)_j$ from E_t exceeds the rejection thresholds Γ_i, which are projected from U_r onto U_t: $(E_t)_j > \Gamma_i((U_r)_i^T (U_t)_j)^2$. If this condition is met, the activity values of that component are set to zero.

$$(C_t)_{\text{clean}} = (U_t^T T_r)^+_{\text{trunc}} P_t = (U_t^T T_r)^+_{\text{trunc}} U_t^T D_t \tag{2}$$

where B^+ stands for the pseudoinverse of B, the clean data $(D_t)_{\text{clean}}$ is then obtained by projecting $(C_t)_{\text{clean}}$ back using T_r:

$$(D_t)_{\text{clean}} = T_r(U_t^T T_r)^+_{\text{trunc}} U_t^T D_t \quad (3)$$

Calibration primarily presents challenges through two methods: 1) resting-state calibration before addressing motion artifacts[15], which might not effectively capture variations in brain activity across different states, potentially resulting in the loss of crucial physiological data; and 2) real-time calibration, which seeks to identify clean segments by statistically analyzing signal variance and power distributions within the current signal window. However, this real-time method often falters under heavy motion artifacts, struggling to differentiate between true brain activities and variations caused by noise, which may lead to the inclusion of corrupted data in the calibrated output. To overcome these challenges, we integrate IMU data for real-time motion artifact detection, which are then fed into the ASR for calibration. This allows the ASR to learn the motion patterns from the IMU data. We extract features from the 3-axis accelerometer, including the acceleration, the velocity, and their vector sum. We then identify the feature with the highest cross-correlation to each channel of contaminated data as the reference signal. Consequently, during the processing phase, the ASR can distinguish actual motion artifacts, treating them as part of the clean signal while what was previously considered 'clean' may be re-classified as noise.

Additionally, by employing covariance matrices that aggregate data from all channels, the traditional multi-channel ASR method may inadvertently amplify and distribute noise across channels, misinterpreting consistent noise as valid signals. This can lead to re-distribution of noise during calibration and cleaning processes. Therefore, we propose an improvement of the traditional ASR algorithm to perform calibration and cleaning on a single channel. However, applying ASR on a single biological channel with IMU feature as a reference requires adaptations. Typically, ASR uses a covariance matrix $\text{Cov}(D_t)$ to analyze relationships across multiple channels within an EEG array of size $N \times M$ (channels by samples). For single-channel EEG (size $1 \times M$), this matrix approach is inadequate since covariance is meant to compare multiple channels. Attempting to compute $\text{Cov}(D_t)$ for a single channel would yield invalid result, or just variance, lacking the inter-channel comparison necessary for ASR. To address this, Blind Source Separation (BSS) using Stationary Wavelet Transform (SWT) is employed. The process includes:

$$Z_{\text{ref}} = \text{SWT}(D_{\text{ref}}) \quad (4)$$

$$Z_{\text{EEG/EOG/ECG}} = \text{SWT}(D_{\text{EEG/EOG/ECG}}) \quad (5)$$

where D_{ref} is IMU reference data after z-score normalization to reach the same scale as physiological signals, and $D_{\text{EEG/EOG/ECG}}$ is the physiological signal. These transformed data sets are then processed through ASR, tuned for real-time artifact removal:

$$\text{Cov}(Z_{\text{ref}}, Z_{\text{EEG/EOG/ECG}}) = U\Sigma V^T \quad (6)$$

Here, the covariance matrix or a similar metric is established using the transformed IMU feature Z_{ref} to set the thresholds for the ASR calibration dynamically. The ASR then evaluates each window of data:

$$D_{\text{clean}} = \text{ASR}(Z_{\text{ref}}, Z_{\text{EEG/EOG/ECG}}) \quad (7)$$

In this setup, D_{clean} denotes the cleaned EEG/EOG/ECG data obtained by applying the ASR algorithm to the wavelet-transformed data, effectively isolating and removing motion-induced artifacts. This integration of IMU data and SWT within the ASR framework ensures robust enhancement of neurophysiological data quality in dynamic environments.

While the above framework builds upon the established ASR algorithm, it introduces critical contribution that dedicate specifically to single-channel data and dynamically changing artifact profiles. By employing IMU-based calibration, the proposed method leverages instantaneous motion patterns to guide artifact recognition. Unlike traditional ASR, which depends on identifying "clean" reference segments in advance or relies on multi-channel covariance structures, this approach directly employs the IMU signal as a continuously adaptive reference. As a result, the algorithm can better track and adapt to evolving noise conditions in real-time without the need to assume steady baseline conditions.

More importantly, the introduction of SWT-based Blind Source Separation prior to ASR transforms the single-channel signal into a representation that allows the construction of a surrogate covariance-like structure through the integration of IMU data. This addresses the fundamental challenge of ASR on single-channel recordings, where the lack of inter-channel correlations would otherwise limit the applicability of traditional covariance-driven methods. The transformation also confers the ability to highlight features and frequency components that are most sensitive to motion artifacts, ensuring that the ASR algorithm's thresholding and rejection criteria remain both stable and discriminative.

Compared to conventional motion artifact removal techniques, the proposed method offers several key advantages. First, it employs adaptive noise modeling by monitoring real-time IMU signals, allowing the algorithm to continuously recalibrate itself

to the current motion artifacts. This ensures that the cleaning process is context-aware and not limited by static thresholds or pre-defined clean segments. Additionally, the method is uniquely applicable to single-channel data, overcoming the limitations of traditional ASR, which heavily relies on multi-channel covariance structures. By integrating Stationary Wavelet Transform (SWT) and an IMU reference signal, the approach enables effective ASR-based cleaning even in scenarios where multi-channel recordings are unavailable or economically infeasible. Furthermore, the method enhances robustness and flexibility by dynamically updating thresholds based on actual motion characteristics, reducing the risk of misclassifying physiological signals as noise or vice versa. This ensures that subtle yet significant neural signals are preserved while effectively removing disruptive artifacts.

Supplementary Module SM 2: Physiological Framework for Dizziness Quantification

Feature Extraction

Following signal preprocessing and artifact removal, feature extraction is applied to each physiological modality to quantify the body's response to vestibular stimulation. Signals are segmented into overlapping windows with configurable stride settings to ensure temporal resolution and robust data coverage. Table ST 2 summarizes the extracted features across all sensor types.

EEG Features: A total of 108 features are extracted from EEG signals across four channels (O1, O2, C3, and C4). These include:

- Spectral features: Absolute and relative band powers across Theta, Alpha, Beta, and Gamma bands.
- Band power ratios: Metrics such as Alpha/Beta and Alpha/Gamma, which are commonly used to assess cognitive load and arousal.
- Non-linear features: Petrosian Fractal Dimension (PFD)[16] and Higuchi Fractal Dimension (HFD)[17] are computed to evaluate signal complexity and irregularity.
- Hjorth parameters[18] (Activity, Mobility, and Complexity) provide time-domain descriptors of signal dynamics.
- Entropy-based features: Sample Entropy and Lempel-Ziv Complexity (LZC)[19] capture unpredictability and information richness, reflecting alterations in sensory processing due to vestibular input.

ECG Features: Heart rate (HR) and heart rate variability (HRV) metrics are derived to evaluate autonomic nervous system (ANS) activity in response to vestibular stress. These features offer insight into sympathetic-parasympathetic balance and the body's cardiovascular adaptability to motion-induced stimuli.

EOG Features: Eye movement dynamics are quantified through both horizontal and vertical EOG channels. Extracted features include blink rate and statistical moments (mean, variance, skewness, kurtosis), which indicates that EOG features capture coarse oculomotor activity (e.g., blink patterns) but do not provide clinical measures such as VOR or nystagmus analysis.

EDA Features: Electrodermal activity (EDA) signals are analyzed using basic statistical descriptors (mean, variance, skewness, kurtosis) to assess physiological arousal. These features reflect sympathetic nervous system activity and are linked to stress or alertness levels induced by vestibular perturbations.

Feature Selection

To optimize model performance and reduce dimensionality, we employ a two-stage feature selection strategy consisting of L1-regularized feature selection followed by Linear Discriminant Analysis (LDA)[20]. This sequential approach balances model interpretability, computational efficiency, and class separability.

In the first stage, L1-based feature selection (Lasso) is applied to exploit its inherent sparsity-promoting property. By assigning zero weights to irrelevant or weakly correlated features, this method retains only those variables that contribute meaningfully to the model's predictive accuracy. This not only reduces noise in the feature space but also improves model generalization and interpretability.

Following this, LDA is applied to the reduced feature set to further refine the representation and enhance discriminability. As a supervised linear projection technique, LDA reduces dimensionality to (k - 1), where (k) is the number of classes, while maximizing the ratio of between-class variance to within-class variance. This projection aligns features in a lower-dimensional space optimized for distinguishing between dizziness levels.

Together, this two-step approach improves model robustness, simplifies downstream classification tasks, and facilitates clearer visualization of dizziness-related physiological patterns.

Supplementary Module SM 3: Dizziness Onset Detection

Predicting the onset of dizziness before it becomes consciously noticeable is essential for enabling timely and effective intervention. This onset is highly individualized and challenging to pinpoint due to inter-subject variability and the gradual emergence of symptoms. To address this, we developed a probabilistic framework that estimates the likelihood of dizziness onset over time, rather than relying on discrete class labels. This approach leverages ensemble learning models to generate robust probability estimates of belonging to the dizziness class at each time point. Ensemble methods are particularly suited for handling noisy physiological data and capturing subtle signal variations, offering improved generalizability and prediction accuracy.

We begin by grouping features according to their sensor modality (i.e., EEG, EOG, ECG, and EDA) and train separate classifiers for each channel. For each modality, we evaluate multiple models, including Support Vector Machine (SVM), Logistic Regression (LR), Random Forest (RF), and Extreme Gradient Boosting (XGBoost), using k-fold cross-validation. The best-performing model per channel is selected based on accuracy. The final dizziness onset probability is computed as the average of probability scores across all selected channel-wise models. Although the underlying task is framed as a binary classification, "non-dizziness" (class 0) vs. "dizziness" (class 1), our focus lies not in the hard label assignment but in tracking the continuous evolution of dizziness probability over time. This probabilistic trajectory provides a more sensitive and dynamic representation of dizziness emergence, enabling earlier detection and adaptive intervention.

If $P_{c_i}(x)$ represents the probability from model c_i for input x, N is the number of channels, the combined probability $P(x)$ is:

$$P(x) = \frac{1}{N}\sum_{i=1}^{N} P_{c_i}(x), \tag{8}$$

Fig. 6a illustrates the dynamic change in dizziness probability over time during vestibular stimulation. As shown, the probability rises from below 40% to over 80%, followed by fluctuations around 75%, indicating a transition from a low likelihood of dizziness to a fully perceived state. Identifying the onset of dizziness involves capturing the early phase of this transition—specifically, the point at which the probability begins to increase rapidly but before it stabilizes. To characterize this process, we assume a typical trajectory in the probability curve: a rising trend as vestibular stimulation begins to take effect, reflecting the developing sensation of dizziness. To capture this transition with temporal precision, we employ a 10-second sliding window (stride: 1 second) and apply a moving average filter to smooth short-term fluctuations and accentuate the overall trend in the probability signal.

However, two primary challenges arise in practice: (1) The ensemble-derived probability curve can exhibit false rising edges, even in the absence of dizziness, due to noise or inter-model variability. (2) In real-time applications, future data is inaccessible, which limits the ability to retrospectively confirm whether a given upward trend will stabilize, peak, or reverse. To overcome these challenges and enable early detection, ideally during the rising phase and before dizziness becomes subjectively apparent, we introduce a slope-based detection method. This method quantifies the rate of change in dizziness probability using linear regression over a fixed 40-second window. The slope of the fitted line provides a scalar measure of how rapidly the probability increases. The time point corresponding to the maximum positive slope within a stimulation session is then identified as the dizziness onset, as it typically aligns with the steepest part of the probability curve's ascent. Linear regression is performed within each 40-second window to compute local slopes across the time series. By scanning the entire probability curve in overlapping windows, the algorithm identifies the peak slope, which marks the most abrupt transition from non-dizziness to dizziness. This approach avoids reliance on absolute probability thresholds and instead leverages the rate of change, which is more robust to baseline shifts and individual variability.

To further enhance the prediction of dizziness onset, especially in real-time scenarios, we implemented a Long Short-Term Memory (LSTM) neural network capable of modeling sequential dependencies and temporal context[21]. The LSTM was trained using offline-labeled data, where dizziness onset windows were determined based on the slope-based method. Specifically, if the window with the highest slope occurred at time T, we labeled windows T, $T-1$, $T-2$, and $T-3$ as positive samples (class 1), capturing the gradual onset phase. All other windows were labeled as negative samples (class 0), representing the non-dizzy state.

This labeling strategy enables the LSTM model to learn not only the peak transition but also its preceding temporal context, thereby increasing its ability to anticipate onset before it becomes behaviorally evident. Fig. 6c illustrates the effectiveness of this approach, where the highest slope marker aligns with the sharpest increase in dizziness probability, precisely identifying the transition from stable to unstable vestibular states.

By combining ensemble model-based probability tracking, slope analysis, and LSTM-driven temporal learning, this framework provides a physiologically grounded and computationally robust method for early dizziness onset detection. Such early prediction enables proactive interventions to mitigate safety risks and discomfort in real-world applications.

References

1. Hossain, M. S. *et al.* Motion artifacts correction from single-channel eeg and fnirs signals using novel wavelet packet decomposition in combination with canonical correlation analysis. *Sensors* **22**, 3169 (2022).
2. Seok, D., Lee, S., Kim, M., Cho, J. & Kim, C. Motion artifact removal techniques for wearable eeg and ppg sensor systems. *Front. Electron.* **2**, 685513 (2021).
3. Makeig, S., Bell, A., Jung, T.-P. & Sejnowski, T. J. Independent component analysis of electroencephalographic data. *Adv. neural information processing systems* **8** (1995).
4. Winkler, I., Haufe, S. & Tangermann, M. Automatic classification of artifactual ica-components for artifact removal in eeg signals. *Behav. brain functions* **7**, 30 (2011).
5. Roy, V., Shukla, S., Shukla, P. K. & Rawat, P. Gaussian elimination-based novel canonical correlation analysis method for eeg motion artifact removal. *J. healthcare engineering* **2017**, 9674712 (2017).
6. Dhull, S. K., Singh, K. K. *et al.* Eeg artifact removal using canonical correlation analysis and emd-dfa based hybrid denoising approach. *Procedia Comput. Sci.* **218**, 2081–2090 (2023).
7. Ram, M. R., Madhav, K. V., Krishna, E. H., Komalla, N. R. & Reddy, K. A. A novel approach for motion artifact reduction in ppg signals based on as-lms adaptive filter. *IEEE Transactions on Instrumentation Meas.* **61**, 1445–1457 (2011).
8. Correa, A. G., Laciar, E., Patiño, H. & Valentinuzzi, M. Artifact removal from eeg signals using adaptive filters in cascade. In *Journal of Physics: Conference Series*, vol. 90, 012081 (IOP Publishing, 2007).
9. Nayak, A. B. *et al.* An empirical wavelet transform-based approach for motion artifact removal in electroencephalogram signals. *Decis. Anal. J.* (2024).
10. Felja, M., Bencheqroune, A., Karim, M. & Bennis, G. Removing artifacts from eeg signal using wavelet transform and conventional filters. *WSEAS Transactions on Inf. Sci. Appl.* **17**, 177–183 (2020).
11. Aloui, N., Planat-Chrétien, A. & Bonnet, S. Artefact subspace reconstruction for both eeg and fnirs co-registred signals. In *2021 43rd Annual International Conference of the IEEE Engineering in Medicine & Biology Society (EMBC)*, 208–211 (IEEE, 2021).
12. Blum, S., Jacobsen, N. S., Bleichner, M. G. & Debener, S. A riemannian modification of artifact subspace reconstruction for eeg artifact handling. *Front. human neuroscience* **13**, 141 (2019).
13. Kothe, C. A. E. & Jung, T.-P. Artifact removal techniques with signal reconstruction (2016). US Patent App. 14/895,440.
14. Chang, C.-Y., Hsu, S.-H., Pion-Tonachini, L. & Jung, T.-P. Evaluation of artifact subspace reconstruction for automatic artifact components removal in multi-channel eeg recordings. *IEEE transactions on biomedical engineering* **67**, 1114–1121 (2019).
15. Mullen, T. R. *et al.* Real-time neuroimaging and cognitive monitoring using wearable dry eeg. *IEEE transactions on biomedical engineering* **62**, 2553–2567 (2015).
16. Yousefzadeh, F., Jahromi, G. P., Manshadi, E. M. & Hatef, B. The effect of prostration (sajdah) on the prefrontal brain activity: A pilot study. *Basic clinical neuroscience* **10**, 257 (2019).
17. Al-Nuaimi, A. H., Jammeh, E., Sun, L. & Ifeachor, E. Higuchi fractal dimension of the electroencephalogram as a biomarker for early detection of alzheimer's disease. In *2017 39th annual international conference of the IEEE engineering in medicine and biology society (EMBC)*, 2320–2324 (IEEE, 2017).
18. Kaushik, G., Gaur, P., Sharma, R. R. & Pachori, R. B. Eeg signal based seizure detection focused on hjorth parameters from tunable-q wavelet sub-bands. *Biomed. Signal Process. Control.* **76**, 103645 (2022).
19. Ibáñez-Molina, A. J., Iglesias-Parro, S., Soriano, M. F. & Aznarte, J. I. Multiscale lempel–ziv complexity for eeg measures. *Clin. Neurophysiol.* **126**, 541–548 (2015).
20. Tharwat, A., Gaber, T., Ibrahim, A. & Hassanien, A. E. Linear discriminant analysis: A detailed tutorial. *AI communications* **30**, 169–190 (2017).
21. Hochreiter, S. & Schmidhuber, J. Long short-term memory. *Neural computation* **9**, 1735–1780 (1997).

Supplementary Figures

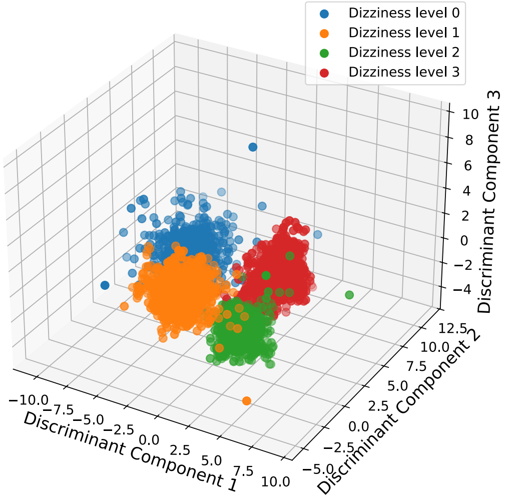

Supplementary Figure SF 1. LDA 4 levels.

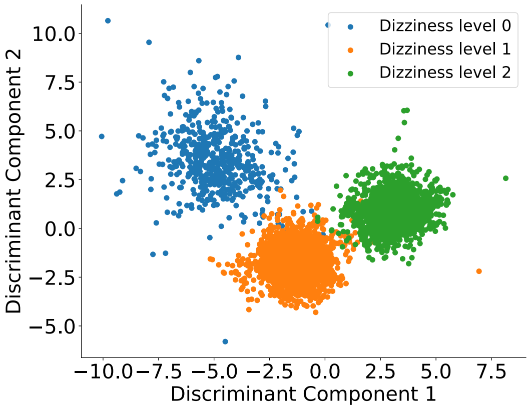

Supplementary Figure SF 2. LDA 3 levels.

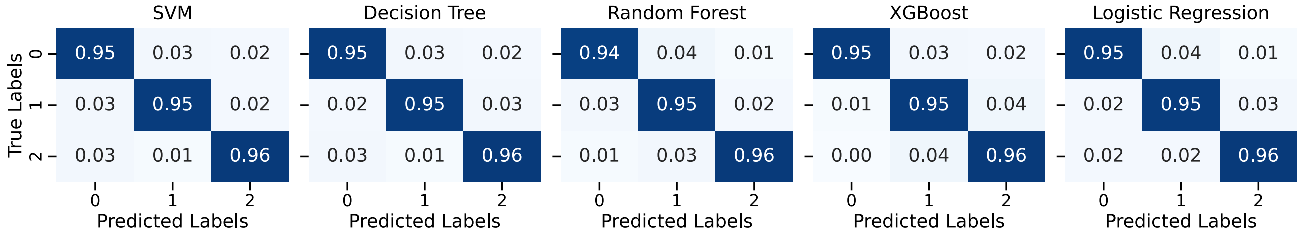

Supplementary Figure SF 3. Confusion matrix for the classification of 3 levels of dizziness.

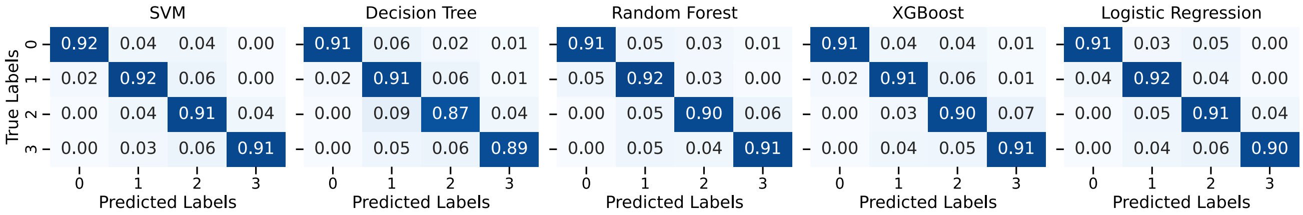

Supplementary Figure SF 4. Confusion matrix for the classification of 4 levels of dizziness.

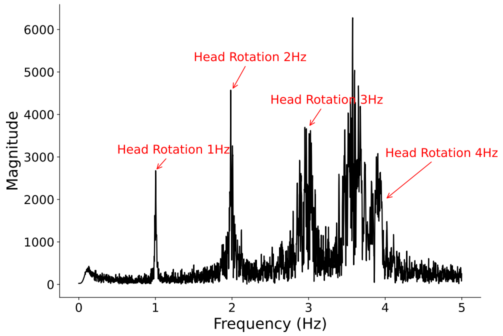

Supplementary Figure SF 5. Frequency from head rotation captured by IMU X-axis.

Supplementary Tables

Supplementary Table ST 1. Performance under different train-test ratios.

Ratio	Accuracy	Precision	Recall	F1-Score
1:1	0.95	0.91	0.93	0.92
1:2	0.93	1.00	0.77	0.87
1:3	0.95	1.00	0.85	0.92

Supplementary Table ST 2. Extracted features from physiological signals.

Signal	Features Extracted
EEG	Absolute Band Power, Relative Band Power, Power Ratio, PFD, HFD, LZC, Hjorth Parameters, Sample Entropy
ECG	Heart Rate (HR), Heart Rate Variability (HRV)
EOG	Blink rate, Mean, Variance, Skewness, Kurtosis
EDA	Mean, Variance, Skewness, Kurtosis
